# Synthesis of 2D anatase TiO_2_ with highly reactive facets by fluorine-free topochemical conversion of 1T-TiS_2_ nanosheets†

**DOI:** 10.1039/d1ta06695a

**Published:** 2022-06-21

**Authors:** Marco Zarattini, Chaochao Dun, Liam H. Isherwood, Alexandre Felten, Jonathan Filippi, Madeleine P. Gordon, Linfei Zhang, Omar Kassem, Xiuju Song, Wenjing Zhang, Robert Ionescu, Jarrid A. Wittkopf, Aliaksandr Baidak, Helen Holder, Carlo Santoro, Alessandro Lavacchi, Jeffrey J. Urban, Cinzia Casiraghi

**Affiliations:** Department of Chemistry, University of Manchester Oxford Road Manchester UK M13 9PL cinzia.casiraghi@manchester.ac.uk; The Molecular Foundry, Lawrence Berkeley National Laboratory Berkeley CA 94720 USA; Dalton Cumbrian Facility, University of Manchester, Westlakes Science and Technology Park Moor Row Cumbria UK CA24 3HA, UK; Physics Department, Université de Namur Rue de Bruxelles Namur Belgium; ICCOM-CNR Via Madonna del Piano 10 50019 Sesto Fiorentino (FI) Italy; Applied Science and Technology Graduate Group, University of California Berkeley CA 94720 USA; School of Automotive Engineering, Guangdong Polytechnic of Science and Technology Zhuhai P. R. China; International Collaborative Laboratory of 2D Materials for Optoelectronics Science and Technology of Ministry of Education, Institute of Microscale Optoelectronics, Shenzhen University Shenzhen 518060 P. R. China; HP Laboratories 1501 Page Mill Road Palo Alto California 94304 USA; Department of Materials Science, University of Milano-Bicocca Via Cozzi 5 20125 Milano Italy

## Abstract

Two-dimensional (2D) anatase titanium dioxide (TiO_2_) is expected to exhibit different properties as compared to anatase nanocrystallites, due to its highly reactive exposed facets. However, access to 2D anatase TiO_2_ is limited by the non-layered nature of the bulk crystal, which does not allow use of top-down chemical exfoliation. Large efforts have been dedicated to the growth of 2D anatase TiO_2_ with high reactive facets by bottom-up approaches, which relies on the use of harmful chemical reagents. Here, we demonstrate a novel fluorine-free strategy based on topochemical conversion of 2D 1T-TiS_2_ for the production of single crystalline 2D anatase TiO_2_, exposing the {001} facet on the top and bottom and {100} at the sides of the nanosheet. The exposure of these faces, with no additional defects or doping, gives rise to a significant activity enhancement in the hydrogen evolution reaction, as compared to commercially available Degussa P25 TiO_2_ nanoparticles. Because of the strong potential of TiO_2_ in many energy-based applications, our topochemical approach offers a low cost, green and mass scalable route for production of highly crystalline anatase TiO_2_ with well controlled and highly reactive exposed facets.

## Abstract 2

Highly crystalline anatase TiO_2_ nanosheets, made by controlled oxidation of TiS_2_ nanosheets, expose the {001} and {100} facets, giving rise to enhancement in HER as compared to Degussa P25 TiO_2_ nanoparticles. No enhancement in ORR was observed.

## Graphical abstract

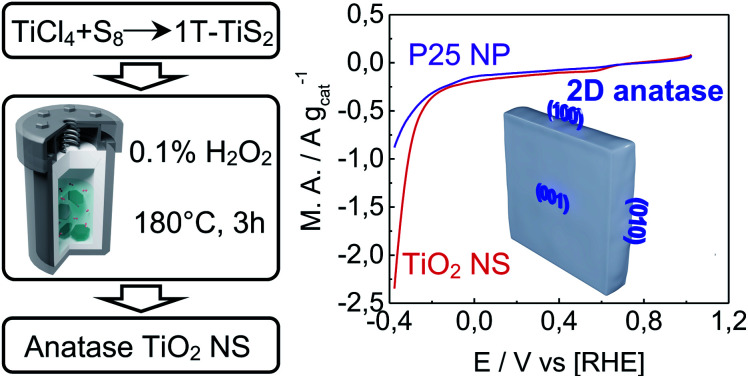

## Introduction

Since the isolation of graphene,^1^ 2D materials have attracted significant interest because of their outstanding properties.^2^ The family of 2D crystals rapidly extended to other materials, typically isolated using micro-mechanical and chemical exfoliation.^3–7^ Despite the intrinsic advantages of these techniques, top-down approaches require layered bulk materials as precursors. However, there are many bulk materials that are not layered and can be made into 2D crystals.^8^ In the past few years, the interest in non-layered 2D nanomaterials, such as metal oxides,^9^ noble metals^10,11^ and metal chalcogenides,^12,13^ has strongly increased due to the novel properties arising from the crystal's dimensionality.

Amongst non-layered materials, TiO_2_ is one of the most renowned material in the family of 2D Transition Metal Oxides because of its outstanding photocatalytic properties, combined to nontoxicity, low cost and environmentally friendly nature.^14,15^ Although TiO_2_ has been investigated since almost 100 years as photo(electro)catalyst,^16^ a recent discovery has sparked new attention on this material: by using specific surface modification, it is possible to enhance or reduce the surface energy of specific facets, leading to the production of 2D anatase TiO_2_ crystals with exposed high-energy facets, such as the {001}, against the thermodynamically stable {101} facets.^17,18^ 2D anatase TiO_2_ crystals with exposed {001} high-energy facets possess characteristic surface configuration with many dangling bonds and abundant surface defects, giving rise to enhanced catalytic properties compared to ordinary TiO_2_ nanocrystallites.^19–22^

The 2D anatase TiO_2_ facet engineering approach is based on the preferential interaction between fluorine ions and the {001} facets of anatase TiO_2_ crystals.^18^ Typical sources of fluorine ions are: hydrofluoric acid,^18^ ammonium bifluoride,^23^ 1-butyl-3-methylimidazolium tetrafluoroborate,^24^ and the titanium tetrafluoride^18^ precursor itself. The use of Fluorine-based compounds makes the process harmful and environmentally unfriendly. Therefore, it is of crucial importance to develop alternative routes able to provide highly crystalline 2D anatase TiO_2_ with high amount of highly reactive exposed facets. Up to now F-free routes did not provide either high crystallinity or pure phase or higher amount of exposed reactive facets and often require time consuming or expensive treatments, such as calcination.^25–36^

Topochemical conversion is widely used in material chemistry, in particular for the synthesis of different types of materials, including several types of 2D crystals.^37–39^ However, it has been rarely applied to produce 2D anatase TiO_2_ with high energy facets. To the best of our knowledge, only one pioneering work has used F-free topochemical conversion, based on a multistep process where layered titanate is first converted into a distorted anatase structure, by exchange of lithium ions and potassium ions with hydrogen, and then this intermediate precursor is converted into crystalline anatase TiO_2_ by hydrothermal or microwave treatment.^40–42^

Herein, we demonstrate a novel, simple, safe, green and low cost F-free strategy to growth single-crystalline 2D anatase TiO_2_ enclosed by high energy {001} and {100} facets, based on the topochemical conversion of 1T-TiS_2_ nanosheets, acting as both the Ti source and sacrificing 2D scaffold, due to its high reactivity to Oxygen.^43,44^ This process does not require intercalation with lithium or potassium to form intermediate precursors, and more important, it enables production of nanosheets with average lateral size of ∼40 nm and thickness of ∼3.8 nm, exposing {001} and {100} facets, in contrast to previous works based on topochemical conversion.

While facet engineering^45,46^ of anatase TiO_2_ has been widely studied in photocatalysis,^47,48^ electrocatalysis has been hardly investigated. In order to evaluate the effect of the exposure of high energy facets, without the introduction of defects or dopants, we tested the electrocatalytic performance of our 2D anatase TiO_2_ in oxygen reduction reaction (ORR) and hydrogen evolution reaction (HER), and we compared the activity with commercial Degussa P25 TiO_2_ nanoparticles. The material exhibits practically no activity towards ORR, while it outperformed the commercial Degussa P25 TiO_2_ and the blank electrode (glassy carbon) as electrocatalyst towards HER, confirming the use of facet engineering to tune the electrocatalytic activity of this material. Our work points out the need to better understand the correlation between structure (types and amount of facets, defects, dopants, addition of other 2D materials *etc*) on the electrocatalytic properties of TiO_2_.

Considering that TiO_2_ is used in many applications, from solar cells to batteries, our method offers a simple and green alternative for the production of 2D TiO_2_ nanosheets exposing high energy facets.

## Results and discussion

### Topochemical conversion of 1T-TiS_2_ nanosheets

**Fig. 1:**
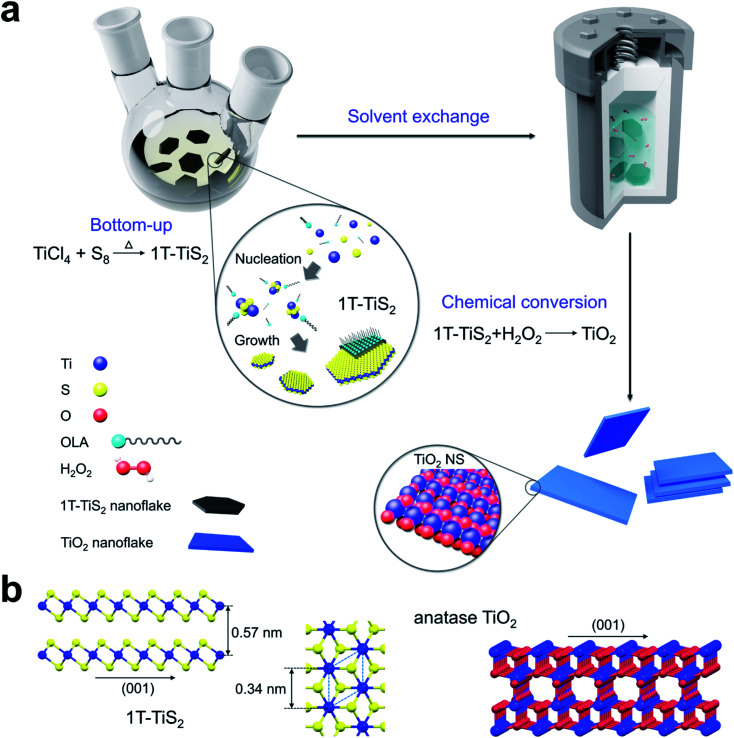
Schematic of the synthetic method of anatase TiO_2_ nanosheets. (a) The process is made of two steps: first, the precursor is made by bottom-up, then the desulfurization process is conducted under controlled hydrothermal conditions. This allows the topochemical conversion of the precursor into anatase TiO_2_ nanosheets, whose structure is depicted in (b). For clarity, Cl atoms are omitted.

Fig. 1 shows a schematic of the synthesis of anatase TiO_2_ nanosheets based on the topochemical conversion of 1T-TiS_2_ nanosheets. In this approach, 1T-TiS_2_ nanosheets are made first through a solution-phase reaction^43^ between titanium tetrachloride (TiCl_4_) and oleylamine–sulfur (OLA : S) complex under inert atmosphere (see methods for further details). Subsequently, the as-synthesized 1T-TiS_2_ nanosheets are washed carefully from the capping agent and re-dispersed in ultra-pure water by solvent exchange using various centrifugation steps. Afterwards, desulfurization is obtained by adding hydrogen peroxide (30% w/w) dropwise during magnetic stirring with a final concentration of 0.1%, followed by hydrothermal treatment in the autoclave to attain anatase TiO_2_ nanosheets. Note that a change in structure, possibly associated to desulfurization of the starting TiS_2_, is clearly visible by the change in color of the starting 1T-TiS_2_ nanosheets from black to white at the end of the process (Fig. S1†).

It is well known that TiS_2_ possesses low oxidative stability under ambient conditions,^43,44^ leading prevalently to amorphous oxide structures of Ti^3+^ and Ti^4+^. In order to achieve high crystallinity, we exploit the fact that desulfurization of TiS_2_ is kinetically promoted by the presence of peroxides species in the dispersion under supercritical conditions^49^ and, at the same time, we make use of 1T-TiS_2_ nanosheets as precursors: being already 2D, 1T-TiS_2_ nanosheets suffer less structural constraints along the out-of-plane direction, compared to bulk TiS_2_ or other precursors, by making the substitution of sulfur atoms more favorable and avoiding the formation of amorphous or polycrystalline domains, ultimately allowing to preserve the 2D morphology. Note also that the use of 1T-TiS_2_ precursors made by bottom up approaches allows to achieve a narrow distribution in size and thickness of the 1T-TiS_2_ nanosheets, as compared to the use of 1T-TiS_2_ made by top-down approaches.

In our process, H_2_O_2_ is easily decomposed to H_2_O and O_2_. Sulphur is pushed away from the 2D scaffold as H_2_S molecules by reacting with the water, while peroxotitanium (Ti^4+^) species will be formed due to the high concentration of oxygen species in solution. The formation of such peroxygen-containing species on the surface of the starting material is expected to slow down the hydrolysis of titanium and at the same time to preserve the 2D morphology. Indeed, without the presence of H_2_O_2_ in the pressure vessel the oxidation is uncontrollable, disrupting the sheet morphology and showing an oxide growth behavior in all directions without specific faceting (see comparative characterization of the material produced with and without oxidizing agent in the ESI, Sections 1 and 2†).

Alternative oxidizing agents, such as H_2_SO_4_ and HNO_3_ are likely to lead to the formation of aqueous titanium species promoting the dissolution of Ti and S in the solvent. For example, products of the reaction with H_2_SO_4_ are typically Ti(OH)_3_HSO_4_, Ti(OH)^2+^, Ti(OH)2HSO^4+^. Moreover, the presence of sulphate species can also promote the polymerization of these titanium complexes. Similarly, using HNO_3_ can lead to the formation of Ti(NO_3_)_2_(OH)_2_. Therefore, although other oxidizing agents can turn TiS_2_ to other titanium species containing oxygen, it is unlikely that they will be able to provide such high control over the conversion process to pure anatase phase without introducing unwanted species, or even promoting the formation of low energy facets.

In order to verify the selective conversion of 1T-TiS_2_ nanosheets into anatase TiO_2_ nanosheets, we conducted a comparative analysis on the structural properties of the starting 1T-TiS_2_ and as-produced TiO_2_ nanosheets, synthesized at different reaction conditions. We found that temperature and reaction time in the autoclave as well as the use of the oxidative agent are crucial in obtaining high crystallinity and purity (ESI, Section S1†).

### Optical spectroscopy study

The X-ray diffraction (XRD) patterns of the 1T-TiS_2_ nanosheets and as-produced TiO_2_ nanosheets are shown in Fig. S8a in the ESI.† The XRD of 1T-TiS_2_ nanosheets shows sharp reflection peaks at 15.5°, 34.2°, 44.1° and 53.8° 2*θ*, indexed to the (001), (101), (102) and (210) crystallographic planes that perfectly matches those of hexagonal (*P*3̄*m*1) TiS_2_ (JCPDS card no. 88-1967), confirming the purity of the phase and the absence of any oxide by-products.^50^ In addition, 1T-TiS_2_ flakes possess higher reflection intensity ratio, in particular between the (001) and (101) crystallographic planes, indicating that the as-synthesized TiS_2_ nanosheets is a few-layers crystal due to the preferential crystal orientation along the (001) direction of the flakes. These characteristic peaks completely disappear after the topochemical conversion process. The converted nanosheets show XRD peaks at 25.2°, 37.8°, 47.9°, 53.9° and 55° 2*θ*, which correspond to the (101), (004), (200), (105) and (211) planes, respectively. These peaks match the typical XRD pattern observed in pure anatase TiO_2_ with tetragonal phase (JCPDS card no. 21-1272), confirming successful conversion into anatase TiO_2_. It is noteworthy to mention that broadening of the Bragg peaks is observed in the spectrum of 2D anatase TiO_2_, as compared to the starting 2D template, indicating a decrease in the flake's size.

The formation of anatase TiO_2_ with high energy facets obtained by using the oxidizing agent was confirmed by using several optical spectroscopy techniques, such as Raman spectroscopy, UV-Vis absorption spectroscopy and X-ray photoelectron spectroscopy (XPS), Section 2 in the ESI.† The Raman spectrum of the converted TiO_2_ nanosheets shows a number of Raman active modes at 144 cm^−1^, 392 cm^−1^, 511 cm^−1^ and 630 cm^−1^ ascribed to *E*_g_, *B*_1g_, *A*_1g_ + *B*_1g_ and *E*_g_ vibrational modes of pure anatase TiO_2_ phase,^51^ respectively. No additional peaks associated to 1T-TiS_2_ are seen. The UV-Visible spectrum of as prepared 1T-TiS_2_ nanosheets (Fig. S8c, ESI†) shows a broad absorption band centered at 590 nm and some weak absorptions at around 300 nm. In contrast, the UV-Visible spectrum of the converted 2D material shows a strong absorption in the ultraviolet region, confirming the structural change of the precursor. The band gap of our nanosheets, derived from the Tauc plot, is ∼3.5 eV (Fig. S9†), which is larger than the band gap of bulk anatase TiO_2_ (3.2 eV) due to confinement effects, thus confirming the 2D nature of the material produced.

X-Ray Photoelectron Spectroscopy (XPS) was used to investigate the composition and electronic states of the nanosheets (Fig. S8d–f, ESI†). In the case of the 1T-TiS_2_ nanosheets, quadruple peaks of Ti 2p spectra at 457.6 eV (Ti^4+^ 2p_3/2_, T–O), 463.6 eV (Ti^4+^ 2p_1/2_, T–O), 455.3 eV (Ti^4+^ 2p_3/2_, T–S) and 461.4 eV (Ti^4+^ 2p_1/2_, T–S)^43,52^ are unveiled with different intensities, showing the presence of a small amount of oxidation on the flake's surface that is a common phenomenon observed in 1T-TiS_2_ as a result of air contact. In contrast, in the converted product only two peaks, at 458.6 and 464.5 eV, were found, consistent with the binding energies of the TiO_2_ chemical states.^53^ This confirms the complete conversion of the 1T-TiS_2_ into the metal oxide. The deconvolution of the O1s spectra (Fig. S8e, ESI†) in the 1T-TiS_2_ demonstrates that most of the oxygen was in the form of Ti–O–Ti and Ti–O–S (O^2−^); the peak also shows a shoulder at higher binding energy that arises from adsorbed sulfate.^43,52^ The O1s region in 2D anatase TiO_2_ revealed two peaks at 529.9 and 531.6 eV, ascribed to O^2−^ bounded to titanium and hydroxide O–H groups, respectively.^53^ The high-resolution S2p spectra (Fig. S8f†) of 2D 1T-TiS_2_ shows two well-defined peaks at 159.9 eV (S^2−^ 2p_3/2_, S–Ti) and 161.2 eV (S^2−^ 2p_1/2_, S–Ti), which correspond to the binding energies of metal sulfide (S^2−^);^43,52^ these peaks mostly disappeared after the conversion leaving only traces of sulfur. The results obtained by XPS spectrum, XRD and Raman spectroscopy all indicate the formation of TiO_2_ nanosheets with high crystallinity.

### Atomic force microscopy study

**Fig. 2:**
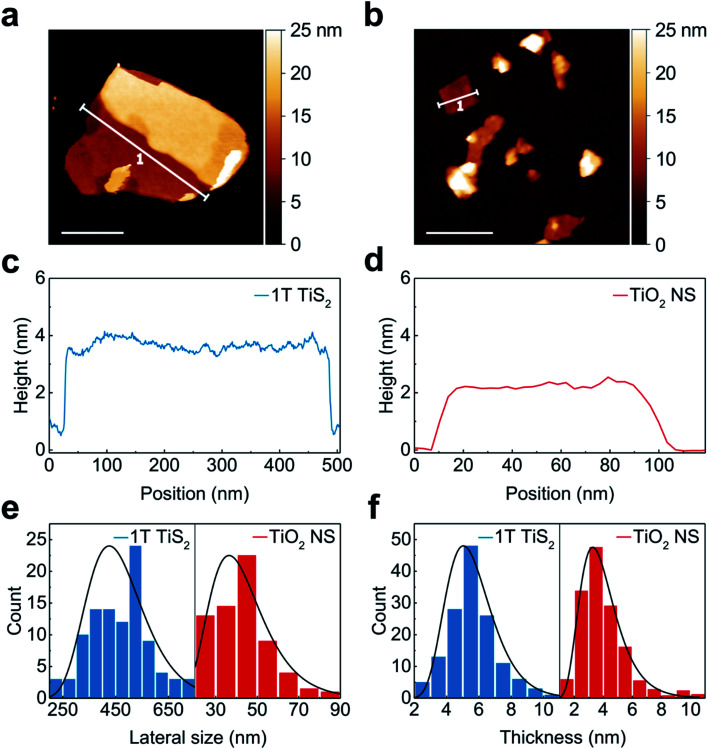
Topographic characterizations of 1T-TiS_2_ and converted anatase TiO_2_ nanosheets. (a and b) AFM pictures of 1T-TiS_2_ and TiO_2_ flakes (scale bar: 200 nm); (c and d) height profile of the flakes selected in panels (a) and (b); (e and f) lateral size and thickness distribution statistics.

The morphology of the 2D nanosheets was examined by atomic force microscopy (AFM). As depicted in Fig. 2a and b, both the as-grown 1T-TIS_2_ and the anatase TiO_2_ nanosheets display clean surfaces (flakes stacking is commonly present in 1T-TiS_2_ samples) and well-defined edges with height profiles of 3.5 nm and 2.3 nm, respectively (Fig. 2c and d). A statistical analysis conducted over 100 flakes shows that 1T-TiS_2_ and TiO_2_ nanosheets have an average thickness of ∼5 nm and ∼3.8 nm, respectively (Fig. 2f), and average lateral dimension of ∼500 nm and ∼40 nm, respectively (Fig. 2e). Hence, the chemical conversion has strongly reduced the lateral size of the nanosheets, in contrast to the thickness. This is probably due to a combination of reaction conditions, such as the gas pressure inside the autoclave vessel, and the chemical potential of H_2_O_2_ under such conditions, as this may promote enough mechanical stress during the desulfurization process and subsequent fracture along the flake's basal plane.

### Electron microscopy study

**Fig. 3:**
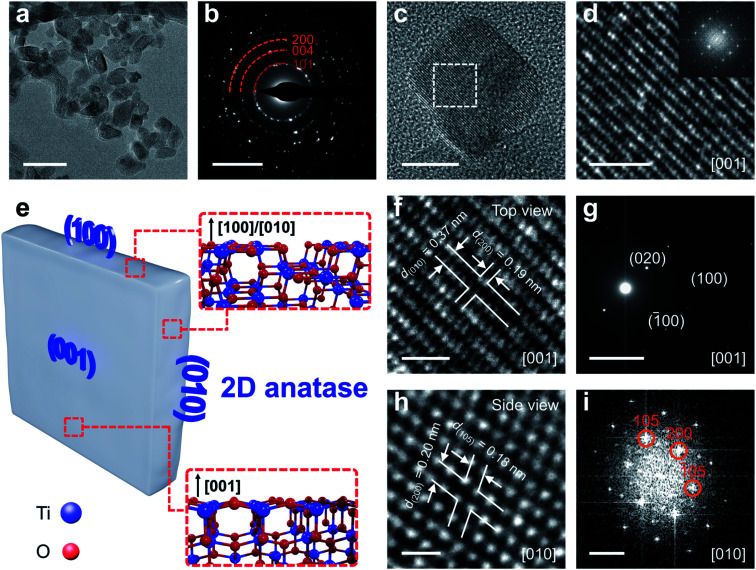
HRTEM characterization of 2D anatase TiO_2_. (a) TEM picture of as-produced anatase TiO_2_ nanosheets (scale bar: 50 nm); (b) SAED of selected area (scale bar: 5 nm^−1^); (c) selected flake (scale bar: 10 nm); (d) magnified HRTEM of the top view (scale bar: 2 nm), inset: corresponding FFT pattern; (e) Schematic of the structure of 2D anatase TiO_2_ exposing (001), (100) and (010) facets (inset: ball and stick models of the corresponding exposed surface); (f) magnified of top view with marked lattice fingers (scale bar: 1 nm); (g) SAED pattern of the corresponding top view (scale bar: 5 nm^−1^); (h and i) magnified HRTEM and corresponding FFT pattern of the side view (scale bar: 0.5 nm and 5 nm^−1^, respectively).

Transmission electron microscopy (TEM) was performed to identify the crystal structure and exposed facets of the crystals obtained. Fig. S13a† shows a representative TEM image of 1T-TiS_2_ nanosheets. Fig. S13b† shows the corresponding selected area electron diffraction (SAED). Fig. 3a shows that the crystal morphology of the product is distinctly different: although the dimensionality is preserved, the flakes are subjected to a downsizing effect, in agreement with the AFM results (Fig. 2e) and the peak broadening observed with XRD measurements. The representative SAED pattern is shown in Fig. 3b: the sequence of diffraction rings is consistent with what is expected for the TiO_2_ anatase phase with lattice spacing (*d*) of ∼0.35 nm, 0.25 nm and 0.18 nm for (101), (004) and (200) crystal planes, respectively. In order to confirm the single-crystalline structure and the exposing facets of the 2D anatase TiO_2_ crystal, high-resolution transmission electron microscope (HRTEM) and correlated SAED were performed. Fig. 3c shows a selected HRTEM image of 2D TiO_2_ flakes with well-defined square-like shape. The SAED and Fast Fourier transform (FFT) pattern of the top facets were measured on isolated 2D anatase TiO_2_ nanosheets (Fig. 3c and Fig. S14 and S15†) as the observation objects. Fig. 3d and f show HRTEM images of selected top faces, exhibiting well-resolved and unabridged lattice fringes. The magnified image depicted in Fig. 3f shows two groups of orthogonal lattice fingers with spacing values of 0.19 and 0.37 nm, in good agreement with the crystal plane spacing (*d*_hkl_) of (200) and (010) of anatase, respectively, thus indicating that the zone axis is [001]. The crystal planes {010} are perpendicular to the zone axis [001] and parallel to the *a*, *b*-plane, Fig. 3e. The SAED pattern also demonstrates the crystal zone axis is along the [001] direction (*i.e.*, out of plane direction) (Fig. 3g), which is perpendicular to the plane (010) in tetragonal systems. The SAED pattern from the flake's top face is a dot matrix instead of concentric rings, which clearly demonstrates the single-crystalline nature of 2D anatase TiO_2_ crystal. Similar results are obtained from the corresponding FFT pattern, as shown in the inset of Fig. 3d (additional HRTEM images with FFT analyses are given in Section S3, ESI†). The same procedure was followed for the side facet. The side facet was analyzed according to the HRTEM images and FFT pattern as shown in Fig. 3h and i. Lattice fringes in two orientations are annotated with the interplanar spacing as 0.20 and 0.18 nm assigned to (200) and (105) planes, in agreement with the XRD and FFT pattern (Fig. 3i). The angle between them is measured to be ∼(68°), very close to the theoretical angle between (105) and (200) planes.^54^ The small angle shift is probably due to bond constrains caused during the desulfurization process. Based on these results, the side facets are determined to be (100) and (010) facets, due to the symmetry of the orthogonal system.

To sum up, these results show that the 2D anatase TiO_2_ single crystals are terminated with {001} and {100} facets, which are high energy facets expected to enhance the catalytic properties of anatase TiO_2_ nanosheets.^19,55^ In particular, the produced 2D anatase TiO_2_ is confirmed to possess the majority of its exposed surface with {001} high energy facets that is the key challenge in facet engineering of anatase TiO_2_.

### Electrocatalytic study

Electrochemical water splitting based on the two half reactions of the hydrogen and oxygen evolution reactions^56^ is a very attractive route for the production of hydrogen as energy carrier. Hydrogen can be also used as fuel in fuel cells to produce electricity where hydrogen is oxidized (hydrogen oxidation reaction, HOR) and oxygen is reduced (oxygen reduction reaction, ORR). However, the high cost and scarcity of the noble metal nanoparticles typically used in the electrocatalysis pose serious challenges in the development of this technology. The cathodic reactions are often sluggish within the electrochemical processes and transition metal (TM) based electrocatalysts are considered attractive alternatives.^57^ In this context, increasing interest for TiO_2_ in electrocatalysis has recently emerged^58^ due to its commercial availability, low cost, non-toxicity, and its high thermal and chemical stability. Furthermore, TiO_2_ is a n-type material with oxygen vacancies that determine its physicochemical properties, enabling to engineer its catalytic properties by modulating the oxygen vacancy density.^59^ So far, only a limited number of studies have focused on the use of TiO_2_ for HER and ORR,^59,60^ while a larger attention has been devoted to the use of TiO_2_ as a support for TM electrocatalysts, and the noble metal nanoparticles.

The use of TiO_2_ in HER and ORR is strongly limited by its low electrical conductivity and poor mass specific activity. Lot of effort is currently spent in trying to reduce the large overpotentials for ORR and HER *via* a boost in the sluggish kinetic activity.^59,61–71^ Careful attention needs to be devoted in those previous cases in which a precious metal or a TM is added, in fact, it must be underlined that TiO_2_ operates as support due to the fact that TMs are much more active compared to TiO_2_ towards electrochemical reactions such as ORR and HER.

**Fig. 4:**
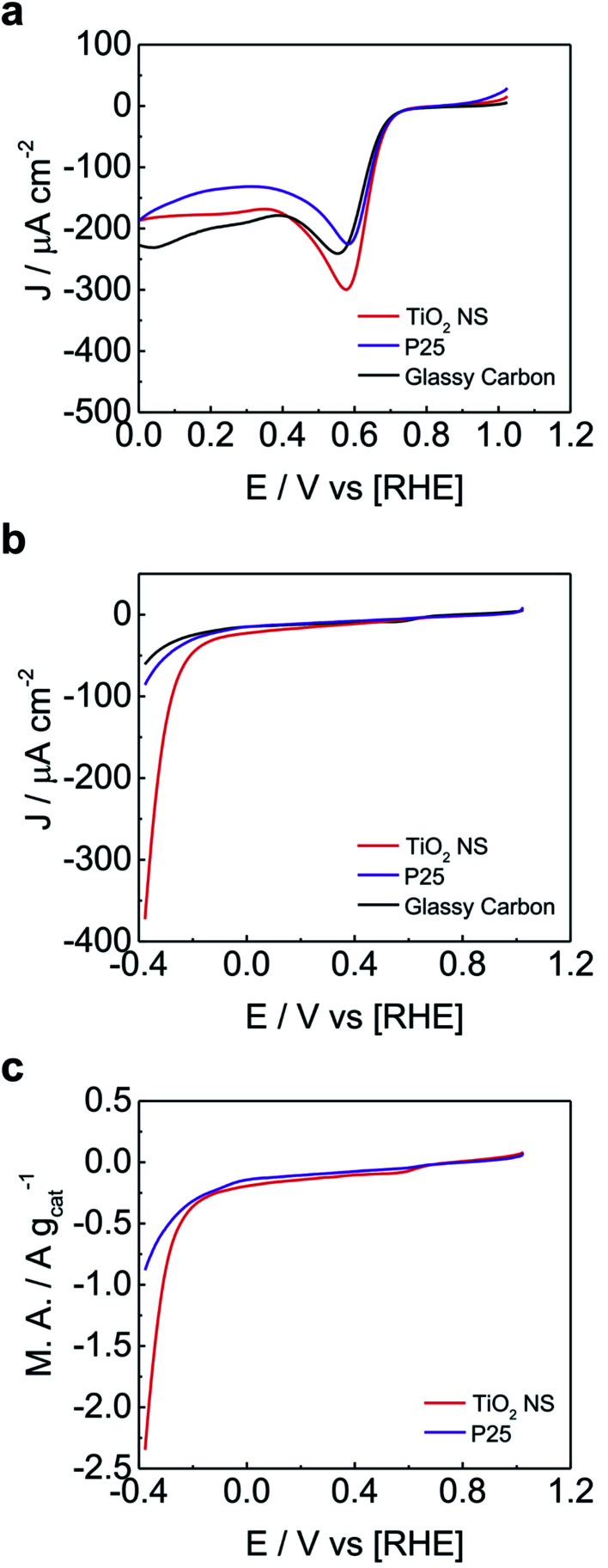
Electrocatalytic study. (a) Oxygen reduction reaction and (b) hydrogen evolution reaction of the 2D anatase TiO_2_ nanosheets, as compared to the commercially available Degussa P25 anatase TiO_2_ nanoparticles and glassy carbon. (c) HER referred to the mass activity for the TiO_2_ nanosheets and P25 nanoparticles.

Facet engineering is a simple approach to tune the electrocatalytic activity of TiO_2_.^19–22^ However, only one work^72^ has investigated the use of anatase TiO_2_ with exposed high energy facets for electrocatalysis. In particular, the TiO_2_ crystals were defective and with relatively low percentage of {001} high energy facets exposed, as compared to 2D anatase TiO_2_ made with F-based approaches, hence it is still unclear if and how much the control of the facets on its own (without additional modifications, such as defects, dopants, *etc*) may affect the electrocatalytic properties of 2D anatase TiO_2._ Therefore, in our study, the as-grown 2D anatase TiO_2_ was investigated for ORR and HER by using a typical three-electrode system using a rotating disk electrode in alkaline environment (0.1 M KOH aqueous electrolyte). An ink of TiO_2_ was deposited over the glassy carbon. This assembly was used as working electrode. All potentials were referred to the reversible hydrogen electrode (RHE). As a benchmark, we also measured a commercially available sample of Degussa P25 anatase TiO_2_ nanoparticles (TiO_2_-NP) at identical loading and under the same experimental conditions. The bare glassy carbon electrode, without the ink deposition of TiO_2_ nanoparticles, was investigated as control experiment for both ORR and HER. The ORR polarization curves are represented in Fig. 4a. The polarization curves demonstrate sluggish ORR activity for commercial anatase TiO_2_ nanoparticles as well as for 2D anatase TiO_2_ crystals. Remarkably, the blank glassy carbon electrode showed similar current to the TiO_2_ commercial and freshly synthesized samples. This fact indicates a low activity towards ORR. This finding is in contrast with ref. 72 where defective TiO_2_ produced with a F-free method was used. As the exposed percentage of high-energy {001} in this material was lower compared to our 2D anatase TiO_2_, our results suggest that the exposure of the high-energy {001} facets on its own is not responsible for the enhanced ORR activity, but this strongly depends on the type and number defects produced in the material synthesis.

Notably, in the case of the HER, the bare glassy carbon electrode showed the lowest activity. This indicates that both the P25 and nanosheets show activity towards HER, Fig. 4b. However, the 2D anatase TiO_2_ nanosheets have remarkably lower overpotentials compared to commercial anatase TiO_2_ nanoparticles, showing enhanced electrocatalytic performance for HER. Note that the two materials show similar BET surface areas (Section S4 ESI† reports all results obtained from the N_2_ physisorption experiments). In order to better show the enhanced electrocatalytic activity of the TiO_2_ nanosheets, the mass activity towards HER is also compared to that of the commercially available Degussa P25 TiO_2_ nanoparticles (Fig. 4c). The synthesized TiO_2_ nanosheets were able to achieve 1 A g^−1^ mass activity at ∼300 mV overpotential, while the Degussa P25 TiO_2_ nanoparticles were able to achieve the same mass activity at ∼400 mV overpotential (Fig. 4c).

HER enhancement was also observed in defective anatase TiO_2_ with exposed {001} facets,^72^ indicating again a complex interplay between high energy facets and defects type and number.

## Conclusions

In summary, this work reports a simple, sustainable and fluorine-free route for the synthesis of ultrathin anatase TiO_2_ nanosheets enclosed exclusively with {001} and {100} high energy facets, which can be exploited for a wide range of applications. Furthermore, our work points out the need to better understand the correlation between the structure (types and amount of facets, defects, dopants, addition of other 2D materials *etc*) and the electrocatalytic properties of TiO_2_ in order to optimize the use of this material in electro-catalysis and allow comparison with TM-based electrocatalysts.

## Experimental

### Materials

Titanium(iv) chloride (TiCl_4_, 99.9%, Acros Organics), sulfur (S, 99.998%, Aldrich), Oleylamine (OLA, technical, 70%, Aldrich), Degussa P25 TiO_2_ nanoparticles, toluene (anhydrous, 99.8%, Aldrich), methanol (anhydrous, 99.8%, Aldrich), hydrogen peroxide (H_2_O_2_, 30%, Aldrich), and ethanol (ACS grade, Aldrich) were used as received without further purification.

### Synthesis of thin 1T-TiS_2_ nanosheets

The synthesis of the precursors is made by using a modified method of the approach reported in ref. 43. In short, 130 mg of elemental sulphur was first dissolved in 5 ml of OLA into a 50 ml three-neck flask. This mixture was then heated to a low-boiling temperature with strong magnetic stirring. After exclusion of air and moisture by back-filling with high-purity argon several times, 0.5 ml of pure TiCl_4_ was injected. It was observed that the solution colour immediately changed from deep orange to black. Following the injection, the temperature of the reaction solution was raised to 300 °C at 10 °C min^−1^. After 3 h, the reaction was rapidly cooled to room temperature, and 1T-TiS_2_ nanosheets were precipitated by centrifugation at 6000 rpm for 20 min and washed up to six times with a mixture of anhydrous toluene/methanol 50 : 50 v/v. Black precipitate was finally re-dispersed in 5 ml of anhydrous ethanol and placed under inert condition inside an argon filled glove-box prior to the conversion.

### Conversion from 1T-TiS_2_ nanosheets to anatase TiO_2_ nanosheets

As-produced 1T-TiS_2_ nanosheets were subjected to solvent-exchange process with pure water by centrifugations. Afterwards, the 1T-TiS_2_ nanosheets were re-dispersed in 15 ml of water and placed under magnetic stirring at ambient conditions. Hydrogen peroxide was added, by achieving a final concentration in the dispersion between 0.1 and 5%. After 10 min, the dispersion was sealed into a 25 ml Teflon-lined stainless autoclave. Subsequently, the autoclave was statically kept at 180 °C for 3 h, and then naturally cooled to room temperature. The mixture was repeatedly centrifuged with water and ethanol, and the white product was collected and dried at 80 °C under vacuum overnight. During the reaction screening, the concentration of H_2_O_2_ in the pressure vessel was set at 0.1%. In order to optimize the reaction, a set of experiments were conducted with reaction times of 1 h, 3 h, 6 h and 12 h, and with and without the presence of H_2_O_2_ in the pressure vessel (Section S1, ESI†).

### Powder XRD

The powders of the precursor and product were carefully dried before each measurement and poured on a zero-background Silicon sample holder. X-ray diffractograms were acquired on a PANalytical X’PERT modular powder diffractometer equipped with a Cu source and operating in the reflection scan geometry. Diffraction patterns were collected in the range 10–60° 2*θ* with a step size of 0.02°. Crystal structures and reference diffraction patterns of the 1T-TiS_2_ and anatase TiO_2_ nanosheets were simulated in Mercury software based on ICSD database.

### UV-vis spectroscopy

Optical spectroscopy was performed using a PerkinElmer l-900 UV-Vis-NIR spectrophotometer in 1 cm path quartz cuvettes. Absorption spectra were recorded between 200 and 800 nm with 1 nm spectral resolution.

### Transmission electron microscopy and electron diffraction

An aliquot of 1T-TiS_2_ or anatase TiO_2_ nanosheets dispersion was diluted using propan-2-ol until optically transparent. The crystals were subsequently deposited directly onto lacey carbon film 300 mesh Cu grids (Agar Scientific) and dried under ambient conditions. Bright field imaging and electron diffraction were carried out using a FEI Tecnai G2 20 S-TWIN Analytical TEM operating at 200 kV. 1T-TiS2 and anatase TiO_2_ lattice spacings were obtained *via* analysis of diffraction patterns using Gatan Digital Micrograph software. HRTEM images were acquired on a JEOL 2100-F microscope with a field-emission gun operated at 200 kV accelerating voltage providing direct images of the atomic structure as well as the selected area electron diffraction characterization of 2D TiO_2_ samples.

### Atomic force microscopy

A Bruker Atomic Force Microscope (MultiMode 8) in Peak Force Tapping mode, equipped with ScanAsyst-Air tips is used to determine the lateral size and thickness distribution of the flakes. The sample was prepared by drop casting the solution on a clean silicon substrate; several areas of 20 μm^2^ were scanned and about 100 flakes were selected for lateral size analysis. Lateral dimension and thickness distributions of 1T-TiS_2_ and anatase TiO_2_ nanosheets were carried out using Gwyddion data processing software.

### Raman spectroscopy

Individual Raman spectra were measured in backscattering geometry under ambient conditions using a Renishaw InVia spectrometer equipped with a 100× objective lens and 2400 lines mm^−1^ grating, resulting in a resolution of ∼1 cm^−1^. A laser of 514.5 nm wavelength was operated below 0.2 mW power for all measurements to avoid damage or local heating effects.

### X-ray photoelectron spectroscopy

The measurements were performed using an Escalab 250Xi spectrometer from Thermo Scientific. The photon source was a monochromatized Al K α line (*hν* = 1486.6 eV). The spectra were acquired using a spot size of 300 μm and constant pass energy (150 eV for survey and 20 eV for high resolution spectra). A flood gun with combined electrons and low energy Ar ions is used during the measurements. A vacuum transfer vessel was used to protect the TiS_2_ nanosheets sample from degradation prior the measurements. All spectra were fitted and weighted with Gaussian Lorentzian function using CasaXPS software.

### Electrochemical characterization

The TiO_2_ dispersions were prepared by dispersing 2.0 mg of TiO_2_ in 2.0 ml of 2-propanol and by sonicating for ≈1 h, following ref. 72 After sonication, 20 μL of the dispersion were deposited on glassy carbon (*A* = 0.1963 cm^2^), which was selected as electrode. Note that because the HER performance of TiO_2_ is not comparable to state of art materials, the choice of the electrode material is very important as this also contributes to the electrochemistry and can overcome the signal produced by TiO_2_. The blank electrode signal must be always recorded when analysing the electrocatalytic properties of new materials as control and background current produced.

The disk electrode was left in contact to the atmosphere till the solvent was fully evaporated. The deposited material was naturally dried at room temperature. The catalyst ink covered the entire surface area of the glassy carbon and had a loading of 0.1 mg cm^−2^ (based on the mass of TiO_2_). After drying, 5 μl of 0.05% Nafion solution in 2-propanol was dropped on the disk as binder. All electrochemical characterizations were conducted using a ParStat 2273 (Princeton Applied Research) and a glassy carbon electrode (0.1963 cm^2^) without rotation. All electrochemical tests were performed in a conventional three-electrode system. Ag/AgCl (in saturated aq. KCl) electrode was used as the reference electrode and a platinum wire (flame annealed prior to the experiment) was used as the counter electrode. 0.1 M KOH aqueous solutions were used as the alkaline electrolyte. All polarization curves run for ORR and HER were recorded at a scan rate of 10.0 mV s^−1^. All the potentials reported in this work were normalized against that of the reversible hydrogen electrode (RHE) using equation *E*_RHE_ = *E*_Ag/AgCl_ + 0.197 V + 0.059 pH for both ORR and HER.

ORR was evaluated in alkaline electrolyte fully saturated of pure oxygen (at least 20 minutes) scanning from +1.022 V *vs.* RHE to −0.278 V *vs.* RHE. HER was instead evaluated from +0.40 V *vs.* RHE to −0.40 V *vs.* RHE. HER tests were conducted flushing pure nitrogen gas into the liquid electrolyte for at least 20 minutes in order to removing the oxygen from the electrolyte.

Specific surface area values were determined by the Brunauer–Emmett–Teller (BET) method by performing N_2_ physisorption at 77 K with a Micromeritics ASAP 2020 instrument.

## Conflicts of interest

Thee are no conflicts of interest to declare.

## Supplementary Material

TA-010-D1TA06695A-s001

## References

[cit1] Novoselov K. S. (2005). *et al.*, Two-dimensional atomic crystals. Proc. Natl. Acad. Sci. U. S. A..

[cit2] Novoselov K. S. (2012). *et al.*, A roadmap for graphene. Nature.

[cit3] Butler S. Z. (2013). *et al.*, Progress, Challenges, and Opportunities in Two-Dimensional Materials Beyond Graphene. ACS Nano.

[cit4] Hu C. (2020). *et al.*, Dispersant-assisted liquid-phase exfoliation of 2D materials beyond graphene. Nanoscale.

[cit5] Niu L. (2016). *et al.*, Production of Two-Dimensional Nanomaterials via Liquid-Based Direct Exfoliation. Small.

[cit6] Das S. (2015). *et al.*, Beyond Graphene: Progress in Novel Two-Dimensional Materials and van der Waals Solids. Annu. Rev. Mater. Res..

[cit7] Zhang H., Cheng H. M., Ye P. (2018). 2D nanomaterials: beyond graphene and transition metal dichalcogenides. Chem. Soc. Rev..

[cit8] Tan C., Zhang H. (2015). Wet-chemical synthesis and applications of non-layered structured two-dimensional nanomaterials. Nat. Commun..

[cit9] Sun Z. (2014). *et al.*, Generalized self-assembly of scalable two-dimensional transition metal oxide nanosheets. Nat. Commun..

[cit10] Huang X. (2011). *et al.*, Synthesis of hexagonal close-packed gold nanostructures. Nat. Commun..

[cit11] Yin X. (2014). *et al.*, Hanoi Tower-like Multilayered Ultrathin Palladium Nanosheets. Nano Lett..

[cit12] Schliehe C. (2010). *et al.*, Ultrathin PbS Sheets by Two-Dimensional Oriented Attachment. Science.

[cit13] Sun Y. (2012). *et al.*, Fabrication of flexible and freestanding zinc chalcogenide single layers. Nat. Commun..

[cit14] Chen X., Mao S. S. (2007). Titanium dioxide nanomaterials: Synthesis, properties, modifications, and applications. Chem. Rev..

[cit15] Fujishima A., Honda K. (1972). Electrochemical Photolysis of Water at a Semiconductor Electrode. Nature.

[cit16] Goodeve C. F., Kitchener J. A. (1938). Photosensitisation by Titanium Dioxide. J. Chem. Soc., Faraday Trans..

[cit17] Pan G., Liu G., Lu G. Q., Cheng H. M. (2011). On the True Photoreactivity Order of {001}, {010}, and {101} Facets of Anatase TiO2 Crystals. Angew. Chem., Int. Ed..

[cit18] Yang H. G. (2008). *et al.*, Anatase TiO_2_ single crystals with a large percentage of reactive facets. Nature.

[cit19] Lazzeri M., Vittadini A., Selloni A. (2001). Structure and energetics of stoichometric TiO_2_ anatase surfaces. Phys. Rev. B: Condens. Matter Mater. Phys..

[cit20] Katal R. (2020). *et al.*, A review on the synthesis of various types of anatase TiO_2_ facets and their applications for photocatalysis. Chem. Eng. J..

[cit21] Gong X. Q., Selloni A., Batzill M., Diebold U. (2006). Steps on anatase TiO_2_ (101). Nat. Mater..

[cit22] Han X., Kuang Q., Jin M., Xie Z., Zheng L. (2009). Synthesis of Titania Nanosheets with a High Percentage of Exposed (001) Facets and Related Photocatalytic Properties. J. Am. Chem. Soc..

[cit23] Yu J., Xiang Q., Ran J., Mann S. (2010). One-step hydrothermal fabrication and photocatalytic activity of surface-fluorinated TiO2 hollow microspheres and tabular anatase single micro-crystals with high-energy facets. CrystEngComm.

[cit24] Ding K. (2007). *et al.*, Facile synthesis of high quality TiO_2_ nanocrystals in ionic liquid via a microwave-assisted process. J. Am. Chem. Soc..

[cit25] Zheng Z. (2013). *et al.*, Metallic zinc- assisted synthesis of Ti^3+^ self-doped TiO_2_ with tunable phase composition and visible-light photocatalytic activity. Chem. Commun..

[cit26] Huang T., Qiu D. (2014). One-Pot Synthesis of Regular Rhombic Titanium Dioxide Supracolloidal Submicrometer Sheet via Sol-Gel Method. Langmuir.

[cit27] Wang Z. (2012). *et al.*, Crystal facets controlled synthesis of graphene@TiO_2_ nanocomposites by a one-pot hydrothermal process. CrystEngComm.

[cit28] Dinh C. T., Nguyen T. D., Kleitz F., Do T. O. (2009). Shape-Controlled Synthesis of Highly Crystalline Titania Nanocrystals. ACS Nano.

[cit29] Wang L., Zang L., Zhao J., Wang C. (2012). Green synthesis of shape-defined anatase TiO_2_ Nanocrystals wholly exposed with {001} and {001} facets. Chem. Commun..

[cit30] Roy N. (2014). *et al.*, Green Synthesis of Anatase TiO_2_ Nanocrystals with Diverse Shapes and their Exposed Facets-Dependent Photoredox Activity. ACS Appl. Mater. Interfaces.

[cit31] Jiang H. B. (2011). *et al.*, Anatase TiO_2_ Crystals with Exposed High-Index Facets. Angew. Chem..

[cit32] Liang Y. (2020). *et al.*, Ultrathin 2D Mesoporous TiO_2_/rGO Heterostructure for High-Performance Lithium Storage. Small.

[cit33] Majumder D., Roy S. (2017). Non-fluorinated synthesis of anatase TiO_2_ with dominant {001} facets: influence of faceted structures on formaldehyde sensivity. New J. Chem..

[cit34] Ge W. (2020). *et al.*, Ultrafast Response and High Selectivity toward Acetone Vapor Using Hierarchical Structured TiO_2_ Nanosheets. ACS Appl. Mater. Interfaces.

[cit35] Tang X. (2017). *et al.*, Low Temperature Synthesis of Large-Size Anatase TiO_2_ Nanosheets with Enhanced Photocatalytic Activities. Small.

[cit36] Sonker R. K. (2020). *et al.*, Green synthesis of TiO_2_ nanosheet by chemical method for the removal of Rhodamin B from industrial waste. Mater. Sci. Eng., B.

[cit37] Yang S. (2020). *et al.*, Topochemical Synthesis of Two-Dimensional Transition-Metal Phosphides Using Phosphorene Templates. Angew. Chem., Int. Ed..

[cit38] Xiao X., Wang H., Urbankowski P., Gogotsi Y. (2018). Topochemical Synthesis of 2D materials. Chem. Soc. Rev..

[cit39] Tominaka S. (2015). *et al.*, Topochemical conversion of a dense metal-organic framework from a crystalline insulator to an amorphous semiconductor. Chem. Sci..

[cit40] Wen P., Ishikawa Y., Itoh H., Feng Q. (2009). Topotactic Transformation Reaction from Layered Titanate Nanosheets into Anatase Nanocrystals. J. Phys. Chem. C.

[cit41] Wen P., Itoh H., Tang W., Feng Q. (2007). Single Nanocrystals of Anatase-Type TiO_2_ Prepared from Layered Titanate Nanosheets: Formation Mechanism and Characterization of Surface Properties. Langmuir.

[cit42] Chen C. (2014). *et al.*, Microwave-Assisted Topochemical Conversion of Layered Titanate Nanosheets to {010}-Faceted Anatase Nanocrystals for High Performance Photocatalysts and Dye-Sensitized. Sol. Cells.

[cit43] Park K. H. (2008). *et al.*, Unstable Single-Layered Colloidal TiS_2_ Nanodisks. Small.

[cit44] Long E. (2017). *et al.*, An in situ and ex situ TEM study into the oxidation of titanium (IV) sulfide. npj 2D Mater. Appl..

[cit45] Kuang Q. (2014). *et al.*, High-Energy-Surface Engineered Metal Oxide Micro- and Nanocrystallites and Their Applications. Acc. Chem. Res..

[cit46] Zhou Z. Y. (2011). *et al.*, Nanomaterials of high surface energy with exceptional properties in catalysis and energy storage. Chem. Soc. Rev..

[cit47] Wang S., Liu G., Wang L. (2019). Crystal Facet Engineering of Photoelectrodes for Photoelectrochemical Water Splitting. Chem. Rev..

[cit48] Liu G., Yu J. C., Lu G. Q., Cheng H. M. (2011). Crystal facet engineering of semiconductor photocatalysts: motivations, advances and unique properties. Chem. Commun..

[cit49] Ong W. J. (2016). *et al.*, Highly reactive {001} facets of TiO_2_-based composites: synthesis, formation mechanism and characterization. Nanoscale.

[cit50] Lin C. (2013). *et al.*, Hydrogen-Incorporated TiS_2_ Ultrathin Nanosheets with Ultrahigh Conductivity for Stamp-Transferrable Electrodes. J. Am. Chem. Soc..

[cit51] Berger H., Tang H., Lévy F. (1993). Growth and Raman spectroscopy characterization of TiO_2_ anatase single crystals. J. Cryst. Growth.

[cit52] Hu Z. (2019). *et al.*, Ultrathin 2D TiS_2_ Nanosheets for High Capacity and Long-Life Sodium Ion Batteries. Adv. Energy Mater..

[cit53] Jensen H., Soloviev A., Li Z., Søgaard E. G. (2005). XPS and FTIR investigation of the surface properties of different prepared titania nano-powders. Appl. Surf. Sci..

[cit54] Calatayud M., Minot C. (2004). Effect of relaxation on structure and reactivity of anatase (100) and (001) surfaces. Surf. Sci..

[cit55] Liu G. (2014). *et al.*, Titanium Dioxide Crystals with Tailored Facets. Chem. Rev..

[cit56] Huang Z. F. (2017). *et al.*, Design of Efficient Bifunctional Oxygen Reduction/Evolution Electrocatalyst: Recent Advances and Perspectives. Adv. Energy Mater..

[cit57] Wang Y., Li J., Wei Z. (2018). Transition-metal-oxide-based catalysts for the oxygen reduction reaction. J. Mater. Chem. A.

[cit58] Lavacchi A. (2021). *et al.*, Titanium dioxide nanomaterials in electrocatalysis for energy. Curr. Opin. Electrochem..

[cit59] Tominaka S., Ishihara A., Nagai T., Ota K. I. (2017). Nanocrystalline Titanium Oxide Catalysts for Electrochemical Oxygen Reduction Reactions. ACS Omega.

[cit60] Boppella R. (2017). *et al.*, Composite hollow nanosctructures composed of carbon-coated Ti^3+^ self-doped TiO2-reduced graphene oxide as an efficient electrocatalyst for oxygen reduction. J. Mater. Chem. A.

[cit61] Rossmeisl J. (2007). *et al.*, Electrolysis of Water on Oxide Surfaces. J. Electroanal. Chem..

[cit62] Hu W., Chen S., Xia Q. (2014). IrO_2_/Nb-TiO_2_ Electrocatalyst for Oxygen Evolution Reaction in Acidic Medium. Int. J. Hydrogen Energy.

[cit63] Zhao Y. (2016). *et al.*, Ultrafine NiO Nanosheets Stabilized by TiO_2_ from Monolayer NiTi-LDH Precursors: An Active Water Oxidation Electrocatalyst. J. Am. Chem. Soc..

[cit64] Mengyan L., Liu H., Lv T., Ding M. (2018). Synergistic effect of the valence bond environment and exposed crystal facets of the TiO2/SnS2 heterojunction for achieving enhanced electrocatalytic oxygen evolution. J. Mater. Chem. A.

[cit65] Choi Y. K., Seo S. S., Chjo K. H. (1992). Thin Titanium Dioxide Film Electrodes Prepared by Thermal Oxidation. J. Electrochem. Soc..

[cit66] Kim J. H. (2007). *et al.*, Catalytic activity of titanium oxide for oxygen reduction reaction as a non-Platinum catalyst for PEFC. Electrochim. Acta.

[cit67] Boskovic I., Mentus S. V., Pjescic M. (2006). Electrochemical behavior of an Ag/TiO composite surfaces. Electrochim. Acta.

[cit68] Berger T. (2012). *et al.*, The Electrochemistry of Nanostructured Titanium Dioxide Electrodes. ChemPhysChem.

[cit69] Tammeveski K. (1999). *et al.*, The Reduction of Oxygen on Pt-TiO_2_ Coated Ti Electrodes in Alkaline Solution. J. Electrochem. Soc..

[cit70] Mentus S. V. (2004). Oxygen reduction on anodically formed titanium dioxide. Electrochim. Acta.

[cit71] Wang B. (2005). Recent development of non-platinum catalysts for oxygen reduction reaction. J. Power Sources.

[cit72] Pei D. N. (2015). *et al.*, Defective titanium dioxide single crystals exposed by high-energy {001} facets for efficient oxygen reduction. Nat. Commun..

