# Supplementary material for: Synthesis of 2D anatase TiO2 with highly reactive facets by fluorine-free topochemical conversion of 1T-TiS2 nanosheets
Source: J Mater Chem A Mater. 2022 Jun 21;10(26):13884–94. doi: 10.1039/d1ta06695a (PMC9255669; doi:10.1039/d1ta06695a)
Supplement: TA-010-D1TA06695A-s001 [file TA-010-D1TA06695A-s001.pdf]

Electronic Supplementary Material (ESI) for Journal of Materials Chemistry A.
This journal is © The Royal Society of Chemistry 2022

Supporting Information

Synthesis of 2D anatase TiO_2 with Highly Reactive Facets by Fluorine-free Topochemical Conversion of 1T-TiS_2 nanosheets

Marco Zarattini[1], Chaochao Dun[2], Liam H. Isherwood[1,3], Alexandre Felten[4], Jonathan Filippi[5], Madeleine P. Gordon[2,6], Linfei Zhang[7], Xiuju Song[1], Wenjing Zhang[8], Robert Ionescu[9], Jarrid A. Wittkopf[9], Aliaksandr Baidak[1,3], Helen Holder[9], Carlo Santoro[10], Alessandro Lavacchi[5], Jeffrey J. Urban[2] & Cinzia Casiraghi[1]*

[1]Department of Chemistry, University of Manchester, Oxford Road, Manchester, United Kingdom, M13 9PL
[2]The Molecular Foundry, Lawrence Berkeley National Laboratory, Berkeley, CA, 94720, USA
[3]Dalton Cumbrian Facility, University of Manchester, Westlakes Science and Technology Park, Moor Row, Cumbria, United Kingdom, CA24 3HA
[4]Physics Department, Université de Namur, Rue de Bruxelles, Namur, Belgium
[5]ICCOM-CNR, Via Madonna del Piano 10, 50019 Sesto Fiorentino (FI), Italy
[6]Applied Science and Technology Graduate Group, University of California, Berkeley CA, 94720, USA
[7]School of Automotive Engineering, Guangdong Polytechnic of Science and Technology, Zhuhai, P. R. China
[8] International Collaborative Laboratory of 2D Materials for Optoelectronics Science and Technology of Ministry of Education, Institute of Microscale Optoelectronics, Shenzhen University, Shenzhen, 518060, P. R. China
[9]HP Laboratories, 1501 Page Mill Road, Palo Alto, California 94304, United States
[10]Department of Materials Science, University of Milano-Bicocca, Via Cozzi 5, 20125 Milano, Italy

Correspondence and requests for materials should be addressed to C.C. (email: cinzia.casiraghi@manchester.ac.uk)

S1. Topochemical conversion: reaction screening
S2. Spectroscopic characterization
S3. Supplementary TEM pictures
S4. Surface area measurements

S1. Topochemical conversion: reaction screening

In our synthetic approach, 1T-TiS_2 nanosheets, produced by solution-phase reaction, are used as precursors: they are washed carefully from the capping agent and re-dispersed in ultra-pure water by solvent exchange using various centrifugation steps. Afterwards, desulfurization is obtained by adding hydrogen peroxide (30% w/w) dropwise during magnetic stirring with a final concentration of 0.1% followed by hydrothermal treatment in the autoclave to attain the fully converted anatase TiO_2 nanosheets. A characteristic change in colour is observed for the final products (**Figure S1b**), as compared to the precursors (**Figure S1a**).

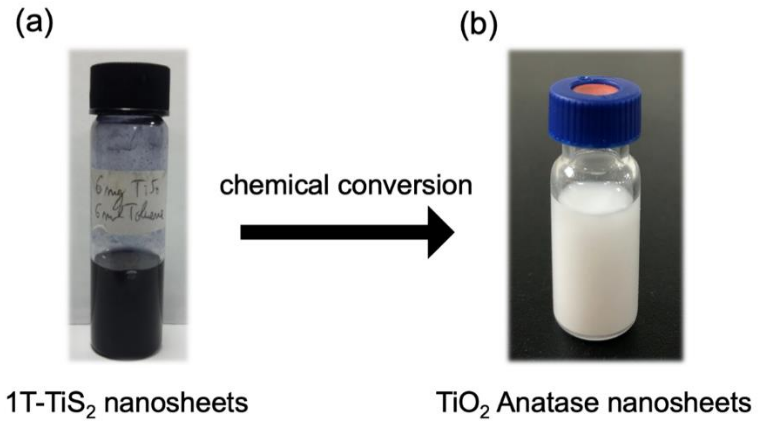

Figure S1: (a) Optical images of 1T-TiS_2 nanosheets precursor and (b) anatase TiO_2 nanosheets dispersions.

To verify the formation mechanism of such enclosed 2D geometry with exposed specific facets of anatase TiO_2 from the chemical conversion of 1T-TiS_2 nanosheets, various combinations of reaction parameters such as temperature and reaction time were tested for the topochemical conversion. **Figure S2 and S3** illustrate the XRD patterns at different stages of the reactions conducted at elevated temperature as 180°C with the presence of hydrogen peroxide (0.1%) and only deionized water, respectively. The structural conversion of 1T-TiS_2 by the substitution of sulfur atoms occurred since after 1 hour of hydrothermal treatment in both cases by the appearance of the anatase pattern and the complete removal of the 1T-TiS_2 crystal reflections. Increasing the reaction time, after 3 hours of hydrothermal process the crystallinity of the nanosheets apparently achieve the maximum. Indeed, the X-ray peak intensities and full- width at half maximum did not change with additional reaction time (i.e. over 9 hours).

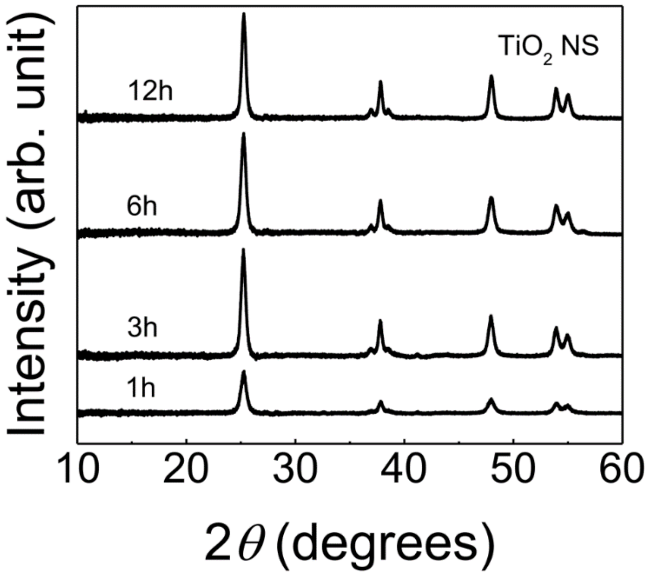

Figure S2: XRD spectra of the material obtained at different time and with H_2O_2 0.1% in the reaction vessel.

Interestingly, the absence of the oxidizing agent leads to much lower crystallinity of the resulting anatase TiO_2 as seen by the difference in the (101) reflex plane in **Figure S4**. Structural evolution from 1T-TiS_2 nanosheets to TiO_2 nanosheets was also tracked by morphological study, further conforming that the topography is clearly affected by the presence of peroxide species.

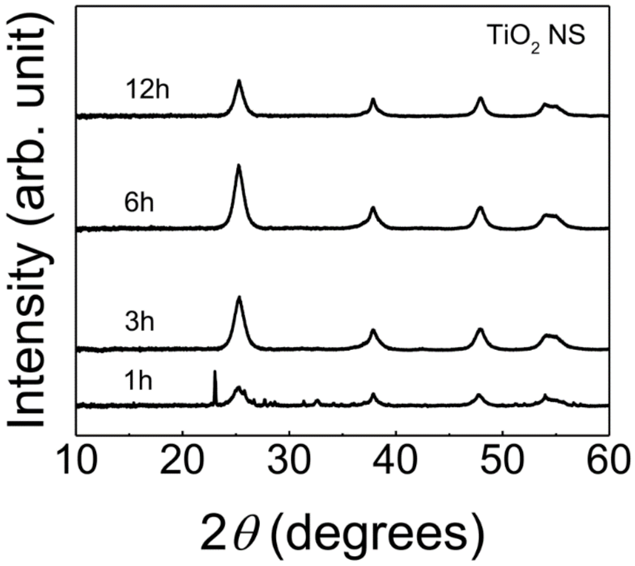

Figure S3: XRD spectra of the material obtained at different times and without the presence of oxidizing reagent in the reaction vessel.

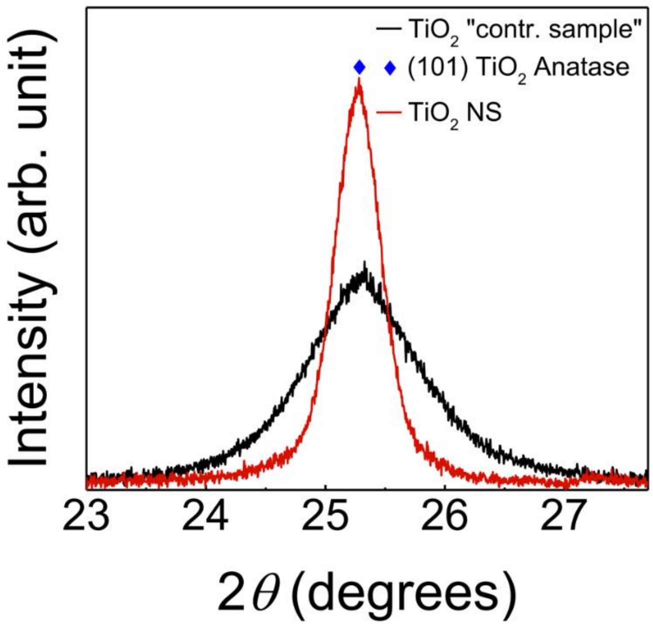

Figure S4: XRD peak at (101) crystal reflection of the TiO_2 crystal obtained without oxidizing agent (contr. Sample) and with oxidizing agent (TiO_2 NS), as compared to the theoretical position of the (101) peak.

Atomic Force Microscopy (AFM) measurements shows that the hydrothermal reaction does not affect the 2D morphology of the template is retain in the first case where both the thicknesses and lateral size are not particularly affected (**Figure S5**). This is in contrast with the crystals obtained without the oxidizing agent, as the 2D morphology is clearly disrupted. **Figure S6** shows a clear growth along the *c*-axis and a markable distortion from the flake shape of the precursor. Without the oxidizing agent, the hydrothermal reaction gives rise to bulky TiO_2 particles with thickness over 200 nm and irregular shapes. It is therefore clear from our results that the involvement of peroxide and radical species (OH^-, $OH^·$) that take parts during the substitution of sulfur atoms during the topochemical conversion have direct influences on the formation of 2D anatase TiO_2 crystals.

Regarding the reaction time or temperature effect, **Figure S7** shows enlarged XRD reflex patterns to show that the relative signal intensities change with increasing autoclaving time or temperature of the sample. The relative intensity of (004) increases compared to (103) and (112) and the corresponding intensity of (211) decreases compared to (105) in both sample at 180°C and 120°C from 1 to 3 hours, respectively. These tendencies were also found in previous reports[2,3] for square or elongated TiO_2 nanocrystals possessing specific exposed facets of anatase phase.

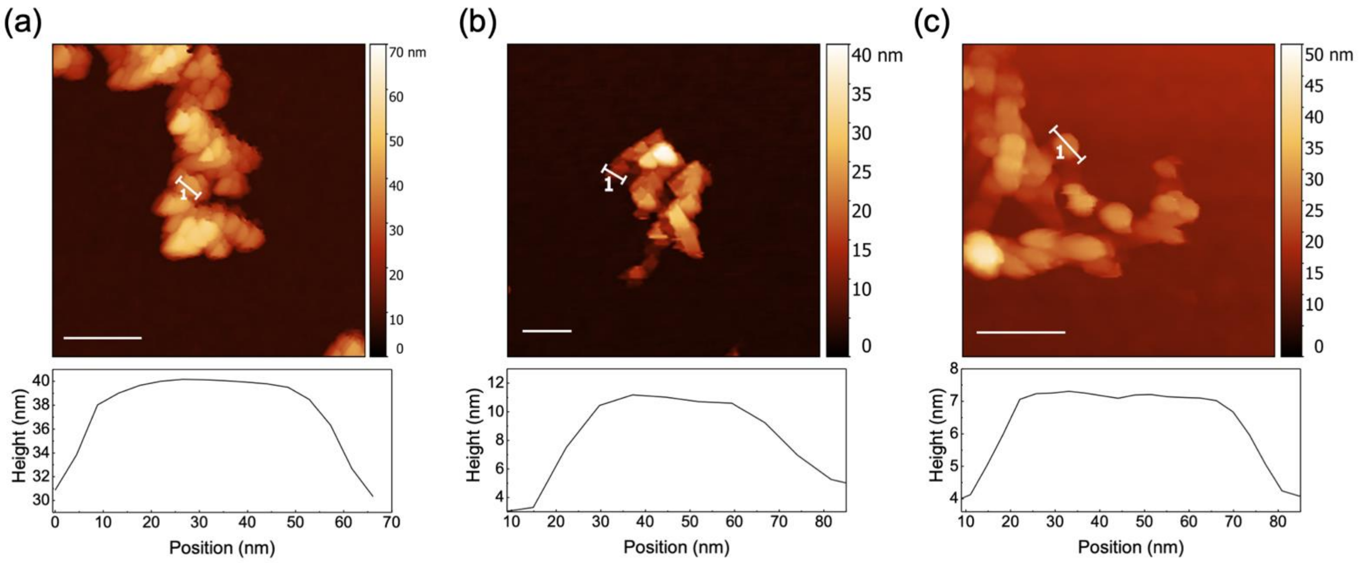

Figure S5: AFM topography and related profiles on crystals obtained (a) after 3h, (b) 6h and (c) 12 h for 0.1% H_2O_2 respectively, at 180°C and using the oxidizing agent (scale bar: 200 nm).

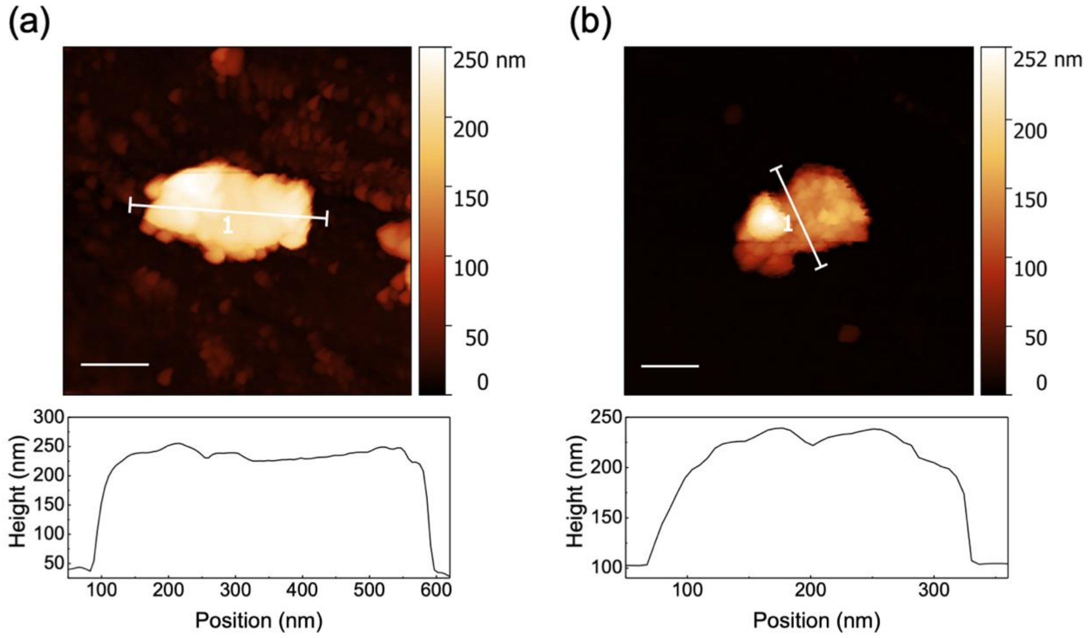

Figure S6: AFM topography and related profiles of crystals obtained: (a) after 3h, (b) after 6h, respectively, at 180°C respectively and without oxidizing agent (scale bar: 200 nm).

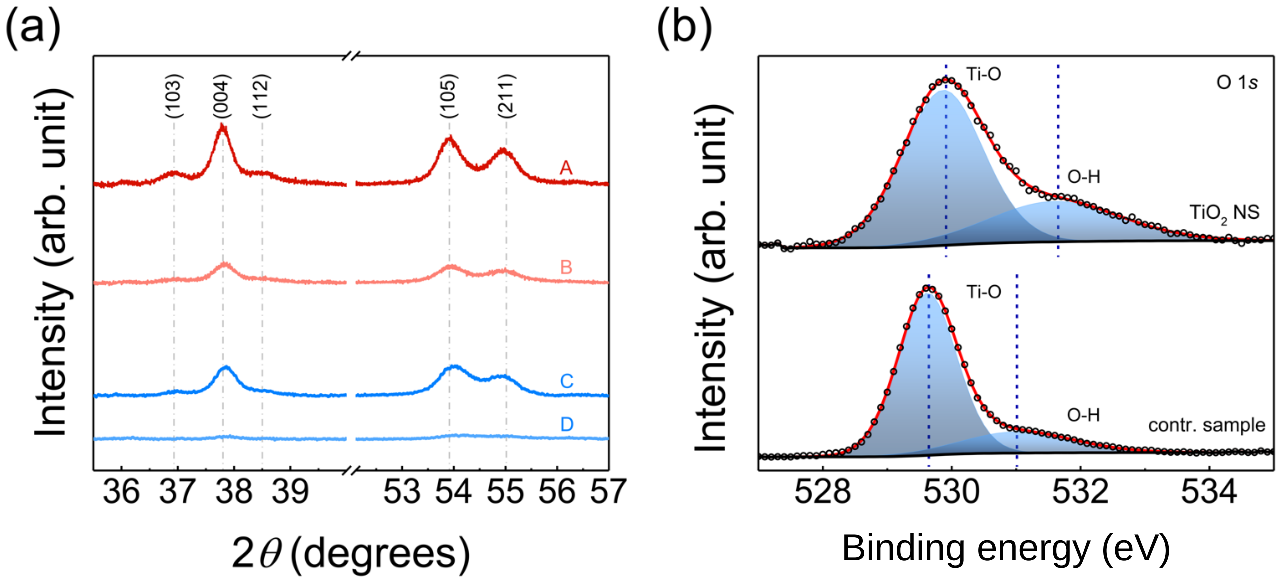

Figure S7: a) Enlarged XRD patterns of the reflexes (103), (004), (112), (105) and (211), labelled A, B samples, obtained at 180°C after 3 and 1h; C, D samples obtained at 120°C after 3 and 1h respectively (obtained with the oxidizing agent); b) high-resolution XPS spectra of O1s region with (NS) and without (contr. sample) the oxidizing reagent.

S2. Spectroscopic characterization

The reaction screening study showed that the highest crystallinity of the 2D anatase TiO_2 crystals is achieved using reaction time of 3h and temperature 180°C. The crystals with such conditions were characterized with a wide range of spectroscopic techniques. Figure S8 shows the results obtained by X-ray diffraction, Raman spectroscopy and UV-Vis spectroscopy. Figure S9 shows the Tauc plot and calculated band gap. Each result is discussed in the main text. Figures S15-S17 shows addition data obtained by X-Ray Photoelectron Spectroscopy. Table S1 summarizes the elemental composition obtained from XPS.

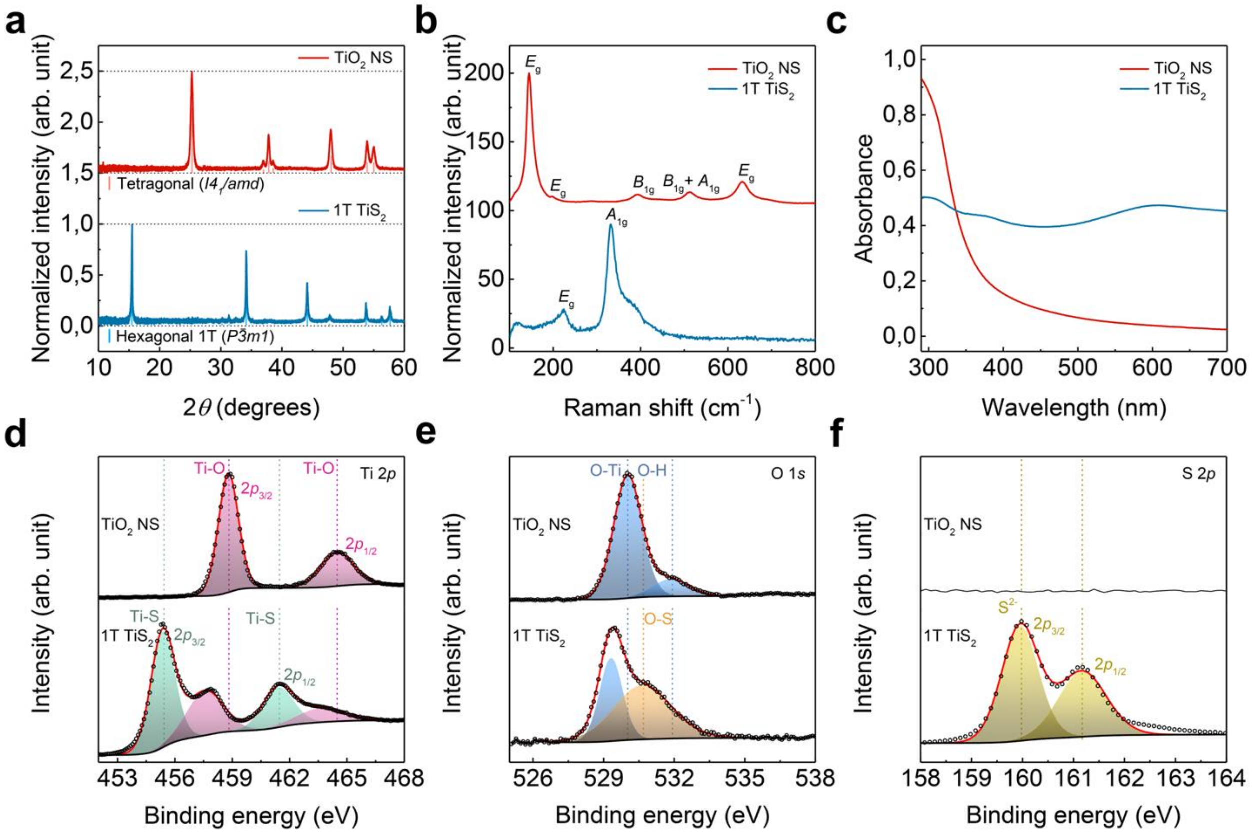

Figure S8: Comparative analysis of 1T-TiS_2 and anatase TiO_2 nanosheets: a) X-ray diffraction patterns; b) Raman spectra; c) Ultraviolet-visible absorption spectra; d-f) X-ray photoemission spectra (XPS) of Ti 2p, O 1s and S 2p core-level electrons respectively

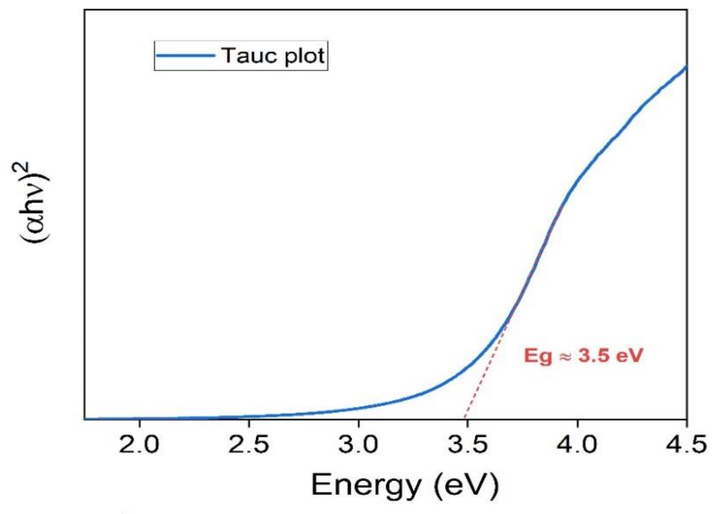

Figure S9: Tauc plot derived from the UV-Vis spectrum of TiO_2 prepared with 0.1% H_2O_2 for 3h.

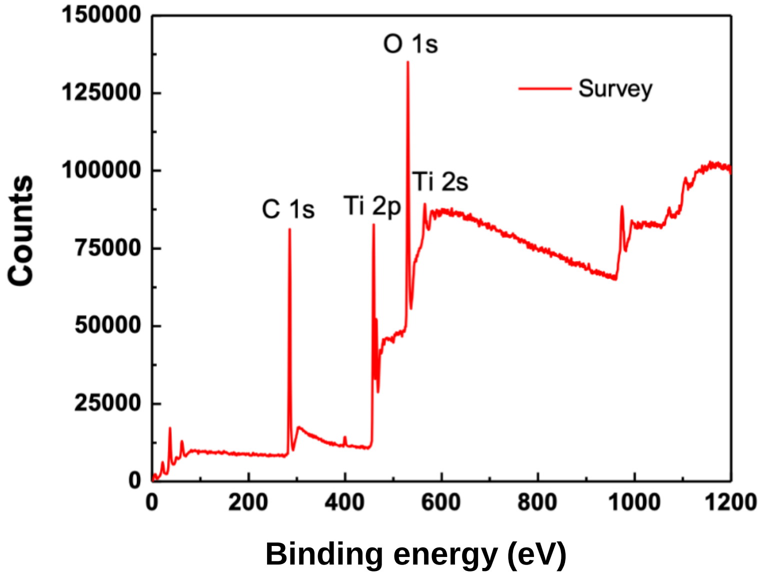

Figure S10: XPS survey of anatase TiO_2 nanosheets, prepared with 0.1% H_2O_2 after 3h.

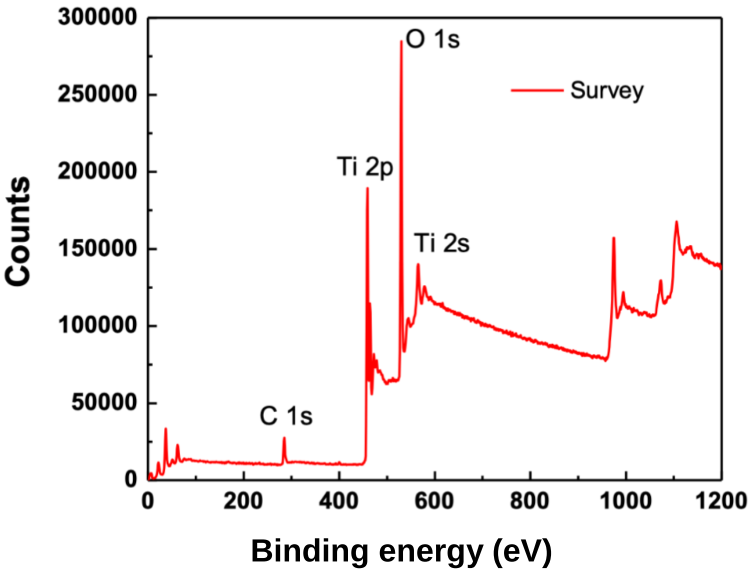

Figure S11: XPS survey of anatase TiO_2, prepared without H_2O_2 after 3h of hydrothermal treatment.

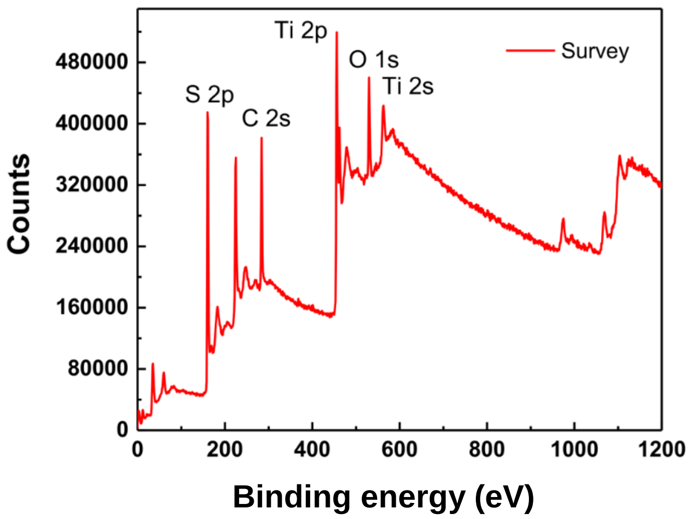

Figure S12: XPS survey of as-produced 1T-TiS_2 nanosheets.

**Anatase TiO_2 nanosheets (3h) /Atomic %**				
**S**	**C**	**N**	**Ti**	**O**
0.44	16.27	0.65	27.77	54.88

**Anatase TiO_2 no ox. Agent (3h) /Atomic %**				
**S**	**C**	**N**	**Ti**	**O**
0.48	14.73	0.60	28.89	55.30

Table S1: element ratio of 2D anatase TiO_2 and 1T-TiS_2 treated without the presence of H_2O_2.

S3. Supplementary TEM pictures

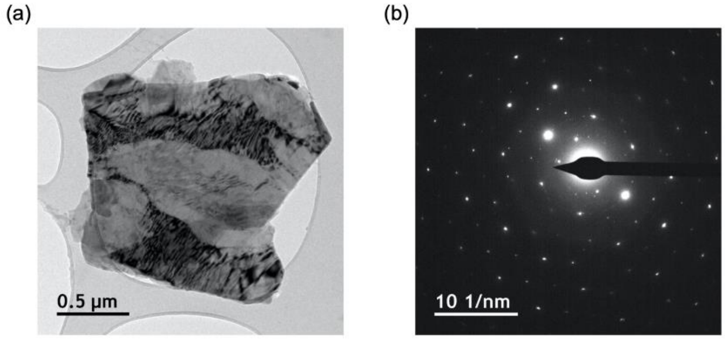

Figure S13: (a) TEM of 1T-TiS_2 nanosheet; (b) corresponding SAED with six-fold pattern characteristics. The nanosheeets are typically made of multiple layers of S-Ti-S stacked on top of each other's comprised in a range of 8 to 15 layers with an average lateral size of ~500 nm and a thickness of ca. 5 nm.

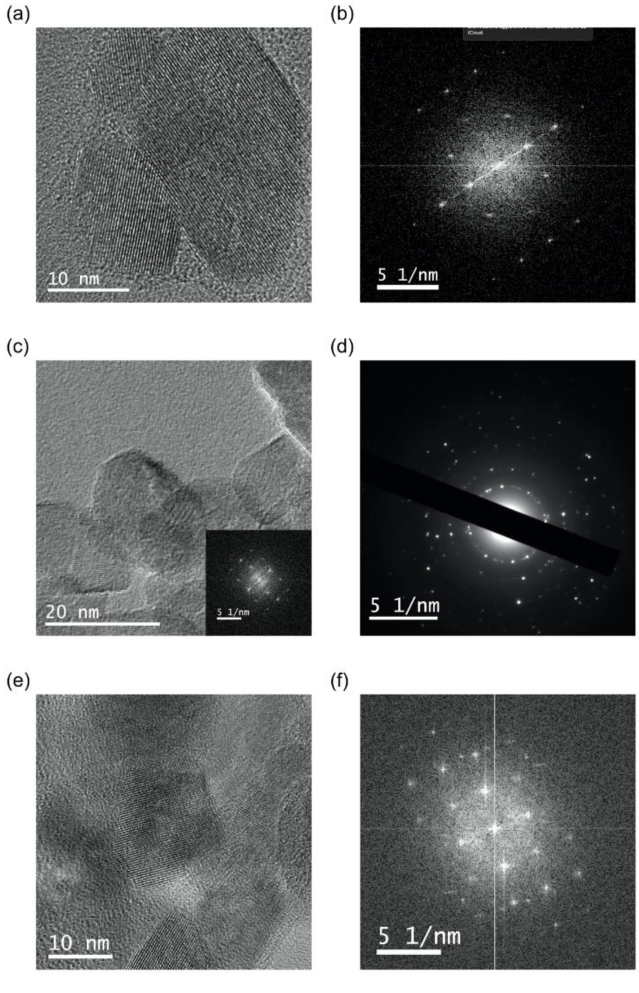

Figure S14: selected HRTEM images of 2D anatase TiO_2 and correlated FFT pattern. SAED pattern is given in panel d, where concentric rings correspond to the anatase phase. Clear square dotted spot is given through generated FFT pattern as shown in panel b and f.

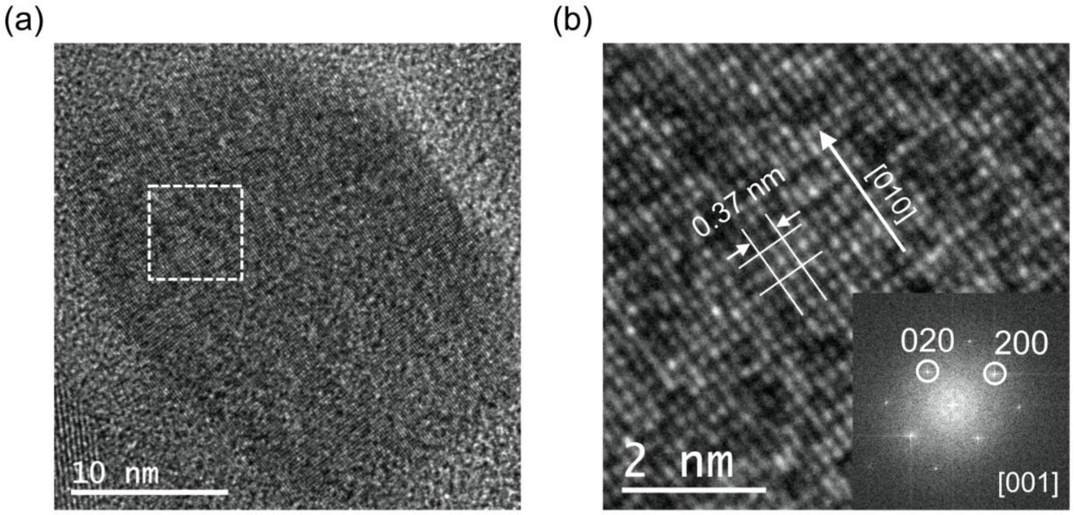

Figure S15: a) selected 2D anatase TiO_2; b) magnified region (marked with white dot line) showing the atomic resolution of the exposed top facet (inset the corresponding FFT pattern): square-like arrangement of the atoms is an indicator of the exposure of (001) anatase facet.

Figures S13-15 show additional images taken by TEM. In particular, in FigurS15b, the unit cell viewed through the [001] crystal orientation is marked with white lines. The generated Fast Fourier Transform pattern (FFT), inset fig. S15b, exhibits a tetragonal atomic arrangement, confirming that the viewed surface is the (001).

Based on the distance between lattice planes d_{hkl} of tetragonal system and the reported unit-cell dimensions of anatase TiO_2,[1] is possible to obtain the calculated $d_{010} = b \approx 0.3785$ nm. The measured lattice fringes with a spacing of $d_{020} = 0.186$ nm, is in well agreement with the theoretical one,[1] thus, giving the expected b distance for the tetragonal system.

From the combination of these characterizations, it can be confirmed that the obtained TiO_2 flakes have square-like surface with exposing {001} facets, and as side facets the corresponding {100} and {010}.

S4. Surface area measurements

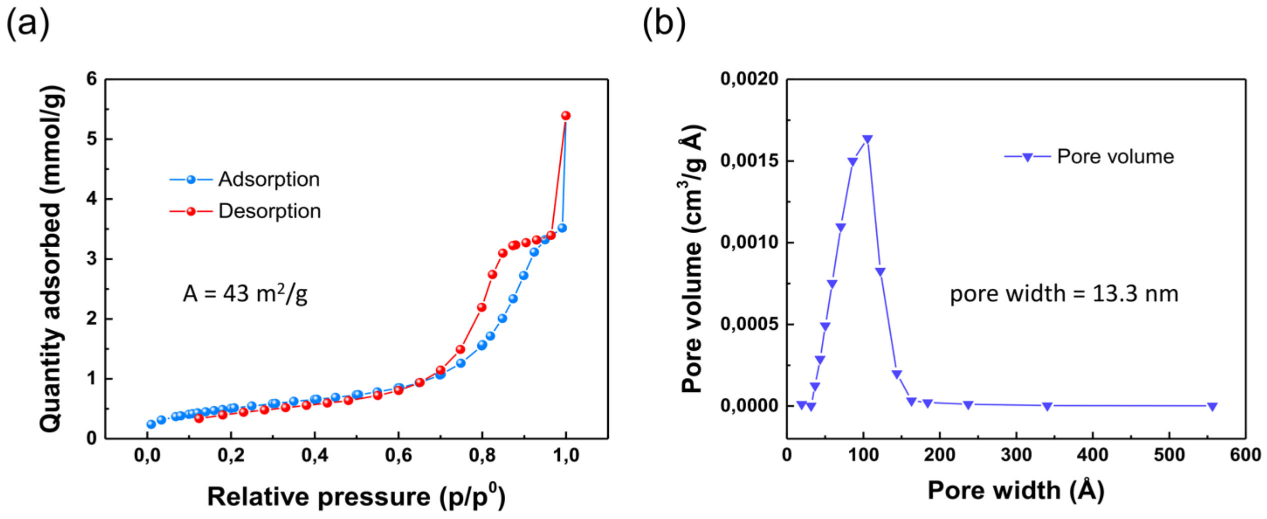

Figure S16: a) Brunauer-Emmett-Teller adsorption isotherm of 2D TiO_2 by using N_2 gas and b) corresponding pore size dimension analysis.

References

1. Burdett, J. K., Hughbanks, T., Miller, G. J., Richardson, J. W. & Smith, J. V. Structural-electronic relationships in inorganic solids: powder neutron diffraction studies of the rutile and anatase polymorphs of titanium dioxide at 15 and 295 K. *J. Am. Chem. Soc.* **109**, 3639-3646 (1987).
2. Dinh, T. et al. Shape-Controlled Synthesis of Highly Crystalline Titania Nanocrystals. *ACS Nano* **3**, 3737-3743 (2009).
3. Cozzoli, P. D., Kornowski, A. & Weller, H. Low-Temeprature Synthesis of Soluble and Processable Organic-Capped Anatase TiO_2 Nanorods. *J. Am. Chem. Soc.* **125**, 14539-14548 (2003).
